# Trust in AI Agent: A Systematic Review of Facial Anthropomorphic Trustworthiness for Social Robot Design

**DOI:** 10.3390/s20185087

**Published:** 2020-09-07

**Authors:** Yao Song, Yan Luximon

**Affiliations:** The Hong Kong Polytechnic University, Kowloon 999077, Hong Kong; yao.song@connect.polyu.hk

**Keywords:** AI agent, human-robot interaction, social robot, facial anthropomorphic trustworthiness, facial features

## Abstract

As an emerging artificial intelligence system, social robot could socially communicate and interact with human beings. Although this area is attracting more and more attention, limited research has tried to systematically summarize potential features that could improve facial anthropomorphic trustworthiness for social robot. Based on the literature from human facial perception, product, and robot face evaluation, this paper systematically reviews, evaluates, and summarizes static facial features, dynamic features, their combinations, and related emotional expressions, shedding light on further exploration of facial anthropomorphic trustworthiness for social robot design.

## 1. Introduction

**Figure 1:**
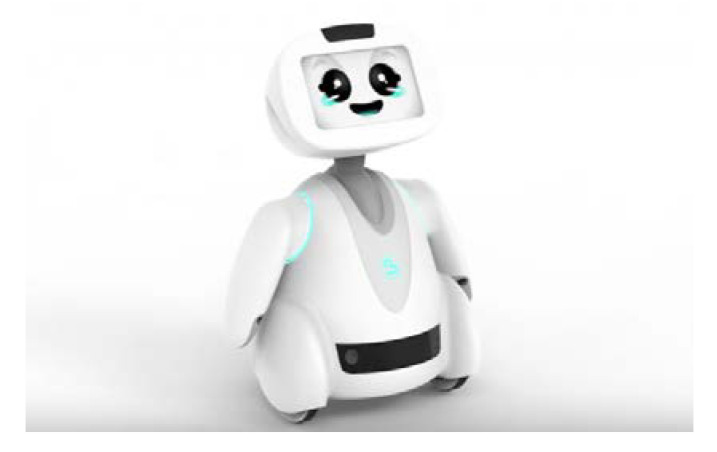
Social robotic product—Buddy Robot^®^.

Since technology has evolved and been applied in different daily contexts [1,2,3,4], the social robot, as one of the representatives of latest innovation, is an artificial intelligence system that could socially communicate and interact with human beings [5,6,7]. Different from traditional humanoid robots (e.g., robotic product, Zora Robot) that are physically embodied with specific human features, some latest social robots (e.g., robotic products, Jibo, Welbo, Misa, QTrobot, Hub, Mykie, and Buddy Robot) are designed with a screen, interfaced with an animated human-like face, to communicate and interact with people [8,9]. For example, Figure 1 shows the Buddy Robot, designed with human-like eyes and mouth, could emotionally accompany and interact with humans, and respond to human needs. Indeed, it might be necessary to design a head-like interface for a social robot to facilitate communication in the human-robot relationship [10,11] since social cognition and perception processes in humans might encourage people to generalize their human-related knowledge and recognition to form an expectation on the behavioral interaction with a social robot [12]. 

Among various characteristics perceptions, such as dominance, friendliness, and attractiveness, trustworthiness towards a social robot plays a crucial role in human-robot interaction (HRI) for two reasons [13]. On the one hand, trustworthiness is crucial in social contexts since it has a significant impact on persuasion and could directly influence people’s intention to follow others’ suggestions [14,15]. On the other hand, social robots work as “communicators and reactors”, providing not only physical help but also emotional support to human, thus they should be initially perceived to be safe to be trusted in [16]. 

Furthermore, Gompei and Umemuro [17] have indicated that there are several issues playing crucial roles in determining trustworthiness in social robots: robot relevant issues (e.g., the characteristics and performance of the robot), human-relevant issues (the specific need, propensity to trust, personality, comfort, self-confidence, attitude, memory, attention, expertise, competency, workload, prior experience, and situation awareness), and scenario relevant issues (task application, task complexity, multi-tasking requirement, physical environment, in-group membership, culture, communication, team collaboration, etc.). Among those, robot-relevant issues are the most significant factors influencing people’s trustworthiness evaluation towards human-robot interaction [18,19]. To be more specific, they might be related to robot behavior, dependability, reliability, predictability, automation, failure rate, transparency, proximity, personality, adaptability, type, and anthropomorphism [18]. For example, prior studies have suggested screened anthropomorphic face tended to make people feel more arousal and more likable [10,20,21], eventually leading to a higher level of perceived trustworthiness for social robots (compared with a mechanical face of social robots) [12].

Indeed, people could evaluate faces on humans and on inanimate objects, such as robots and products, in incredibly limited time [22]. Previous research indicated 100 ms was sufficient for humans to be able to judge multiple personality traits, such as trustworthiness, competence, and aggressiveness [23]. The reason why people seem to be ready to perceive and process faces in objects lie in human evolutionary adaptation: the human face is a salient evolutionary and attention-catching stimuli that would be processed simultaneously [24]. When it comes to evaluating a robot, unlike simply intending to look for the resemblance to a human face, people could perceive concrete facial traits or expressions in robot by aligning particular robot features with human characteristics and making the analogy [10,11,25]. 

Facial features in a social robot might also have an impact on anthropomorphic trustworthiness for such artificial agents [17,19,26]. Previous relevant review papers on trustworthiness focus a lot on summarizing human facial trust features and discussing general trustworthiness in human-computer/human-machine relationship. For example, Hancock et al. [18] evaluate the effects of human, robot, and environmental factors on perceived trust in HRI in general. However, the term of trust is indeed a multi-constructs concept that contained several evaluation stages, such as initial evaluation in the first impression and post-evaluation in latter stages [23]. Besides, although previous literature has tried to assess the role of different facial features in the processing of trust [27], it could not be simply introduced in HRI due to the anthropomorphic nature of social robots [12]. Indeed, research on the facial design of the social robot is a multi-disciplinary field that is rarely systematically analyzed and sporadically studied by various fields. Specifically, there are at least three major disciplinary relating to the facial anthropomorphic trustworthiness of social robots [28]: (1) Since human and social robots might share similar facial features, such as eye and mouth, human facial trustworthiness from psychology, which has long discussed human-specific features on facial trustworthiness [29], might potentially contribute to the knowledge of facial anthropomorphic trustworthiness of social robot. (2) As a robotic product in the commercial market, the social robot might draw inspiration from previous literature on product appearance from marketing and engineering design, which have been discussed on how to build a trustworthy appearance for anthropomorphic products [30]. Anthropomorphic product appearance refers to the physical appearance of a product with human facial traits or features, such as the headlamp of a car, or the power pin of a plug [31]. For example, Maeng and Aggarwal [25] suggested the frontal face of an automobile with lower facial width-height-ratio (fWHR) might enjoy higher trustworthiness evaluation. Although anthropomorphic product design is not directly related to robot facial design, it might also provide, at least, some intuitions for designing a trustworthy robot since they might all share similar anthropomorphic features in communicating trustworthiness. (3) Although previous social robot literature has examined the facial trustworthiness of social robots, it mainly focused on the general effect of the anthropomorphic trustworthiness evaluation, such as the trustworthiness difference between anthropomorphic face and mechanical face in social robots [12]. Indeed, it is a multi-disciplinary research field while single research field could hardly provide specific guidance to help social robot designers and engineers improving trustworthiness on the robot’s face. Although both robot and behavior research has realized the significance of social robot design for its market success and related social benefit for its users [8,22,32], the specific facial features in eliciting the anthropomorphic trustworthiness of social robots still received limited attention. 

Regarding this theoretical and practical research gap, the research question of this study is: 

What are the potential facial features in influencing facial anthropomorphic trustworthiness towards social robots?

To address this research gap, this study tries to present a systematic review of trustworthiness features from the multi-disciplinary fields (human face research, product appearance research, and robot face research), summarize and compare the related theories, and suggest the potential facial features in eliciting anthropomorphic trustworthiness, which contributes to literature on trustworthiness in HRI and sheds light on potential trustworthy facial features in social robot design.

## 2. Methods 

### 2.1. Search terms, Database, and Timespan

As mentioned above, facial trustworthiness features of robot is a multidisciplinary field that should consider its robotic nature from related perspectives: since human and robot share facial structure, human facial trust features could potentially contribute to its facial anthropomorphic trustworthiness; since social robot might be considered as a robotic product, product appearance could potentially contribute to its facial anthropomorphic trustworthiness; robot’s own distinct characteristics, such as anthropomorphism, could also contribute to it. Accordingly, a systematic review of original research paper in English was conducted based on the followed search strings: search strings on facial trustworthiness contained “(face OR facial) AND (trust OR trustworthiness* OR credibility OR trust traits* OR trust features* OR trust signs*)” and strings on product or robot trustworthiness contained “(product OR robot OR anthropomorphism) AND (face OR facial) Trust”. The asterisk * indicates to search both single and plural forms of the keyword. Electronic databases were searched on 1 March, 2019. The databases included Scopus (1960 to March 2019), PsycInfo (1967 to March 2019), and Web of Science (1955 to March 2019). Detailed information was summarized in Table 1. 

### 2.2. Search Strategy 

The search was first narrowed by selecting articles within the subject area of trustworthiness, facial traits, product personality, product evaluation, robot facial trustworthiness, or any other related area of robot trustworthiness design. Articles not in the English language and not original were excluded. Relevant articles were then selected based on four main criteria: (i) the study must have used one or more trustworthiness traits of human face; (ii) the study must have used one or more trustworthiness traits of product appearance; (iii) the study must have used one or more trustworthiness traits of robot “face”; (iv) the study must have used one or more trustworthiness traits of anthropomorphized item.

**Figure 2:**
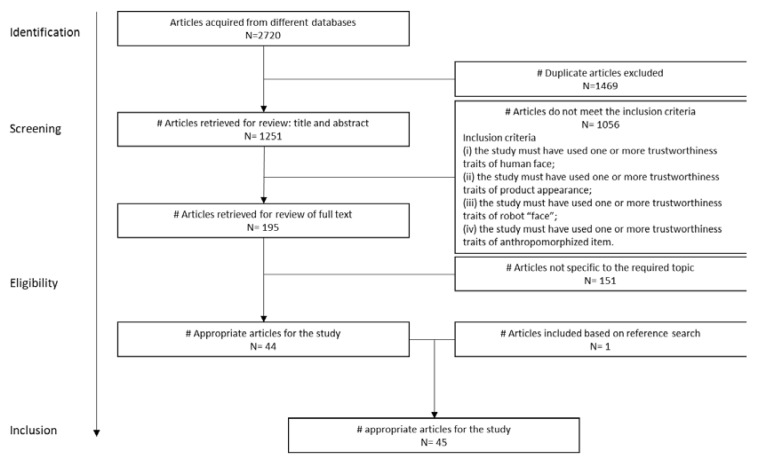
Flow chart of the systematic review process.

The screening of articles was carried out manually in three stages: (A) title screening; (B) abstract screening; and then (C) full-text screening of the related research. The screening criteria at this stage did not retrieve the studies that discussed the general effect of trustworthy face on social judgments, rather than the effect of specific facial features; studies that reported duplicate results, rather than original results; studies that discussed the relationship between facial features and other social judgments, rather than trustworthiness; studies that discussed the characteristics of participants, rather than the specific facial features; studies that explored the neuroscientific explanation of facial trustworthiness. Figure 2 shows the process for this systematic review.

## 3. Results

In total, 2720 published papers (1214 from PsycInfo; 849 from Scopus; and 657 from Web of Science) were potentially identified to be related to this systematic review before the screening process (Figure 1). After excluding the duplicate papers (N = 1469), 1251 papers were then retrieved for the review. Following the screening criteria, 1056 articles were excluded due to their irrelevancy to the current review in the title and abstract screening. Then, 195 research articles were retrieved for full-text screening, out of which 45 are within the inclusion criteria 1 to 4 of this study (Table 2).

## 4. Discussion

The research trend on specific human, product, or robot facial anthropomorphic trustworthy features has been divided into four streams: internal, external, combinations, and emotions (see Table 3). As Calvo et al. and Santos and Young indicated [38,64], the internal features refers to the region containing the eye size, eye color, eye shape, eye gaze, eyebrow, color cues, luminance contrast, cheek, nose, lips, and mouth; the external features refers to the region containing facial width-height ratio (fWHR, refers to a ratio used to determine the width of a person’s face compared to its height), brow-nose-chin ratio, forehead-sellion-nose ratio, hair, forehead, ears, beard, chin, glasses, tattoo, age, and ethnicity; the combinations of different facial features refers to a set of facial features, which make people appear in certain characteristics, such as cuteness, symmetry, and masculine. Dynamic features refer to the movement of specific facial features, while emotional expressions refer to a set of facial features, which activate people to perceive the emotions it evoked.

### 4.1. Implications for Internal Features

The eye region is considered as one of the most significant features that could influence people’s evaluation of trustworthiness, both for human and product [22,52,53,64,72,73,74,75]. This region has several specific attributes that could communicate trustworthiness, such as eye size, eye shape, eye gaze, eye color, and eyebrow [64,67]. Studies on eye shape and size suggest that people with round eyes (vs. narrow) [44,59] and larger eyes (vs. smaller) [53,56] are perceived to be more trustworthy since these characteristics all shared and enjoyed the baby-face appearance traits from an evolutionary perspective [36,58,76]. In addition, eye gaze and eyebrow would also contribute to the people’s credibility. Because eye gaze and related eyebrow are crucial attention-catching cues for social recognition and social interest, the majority research on human facial features suggested that a direct-gaze (vs. looking at others) face with thin (vs. thick) and up-shaped (vs. down) inner ridge eyebrows was anticipated to be not only more trustworthy but also more attractive [52,57,59,64,67,71,77]. In the field of a social robot, there might exist a nuanced relationship between gaze and trustworthiness: Stanton and Stevens [66] suggested constant gaze, compared with averted gaze, might indicate dominance, rather than trustworthiness, and this effect was especially significant when female participants tried to evaluate the robot. As the author mentioned that one of the limitations in their work is the relatively small sample size (N = 52 in three between-subject experiments) and unbalanced gender (N = 14 for male) [66], further research might be necessary to confirm this effect [78]. Unlike other internal features, eye color is not an isolated trait but an ethnic group-related feature, appearing with other facial features within the cultural origin [63]. Although Kleisner and his colleagues [53] mentioned that brown-eyed faces are perceived to be more trustworthy than the blue-eyed faces, they further explained the difference in trustworthiness perception might be related to the facial traits associated.

Nose and mouth region are also perceived to be significant features that have an impact on people’s evaluation of trustworthiness. Prior research has speculated that the central facial properties (nose and mouth region) [61] were significantly positively correlated with attention and trustworthiness. As for the shape of the mouth, there are three types of mouth in the past literature: an upturned mouth (smiling mouth), a downturned mouth (sad mouth), and a neutral mouth [22]. Regarding this, there is a significant difference in the perceived social attributes among these three scenarios: human face or product “facial” appearance with an upturned mouth or a smiling mouth (vs. neutral and downturned) were believed to be more trustworthy, friendlier, and attractive [22,25,33,53]. Cheek, lips, and even teeth could also influence trustworthiness evaluation: people with pronounced cheekbones, wide chins, and thin lips with no-missing front teeth might look more trustworthy than people with shallow cheekbones, thin chins, full lips with missing front teeth [67,69]. When it comes to the nose, previous empirical research shows a contrast result towards the effect of nose attributes on trustworthiness evaluation. While some researchers agreed that a man with a small nose would be perceived as less robust and trustworthy [53], the major literature believed that short nose and shallow nose sellion were significant features for trustworthiness judgments [56,67]. According to evolutionary psychology, people have a strong intention to trust infants whose faces are characterized to have a pug nose [59,71]. The reason for this inconsistency may lie in that Kleisner and his colleagues [53] analyzed the attributes of the nose and its related combinations as a whole (a small nose, chin, and mouth) rather than evaluating the attributes of the nose, separately [71]. 

Various studies have been carried out to explore the effect of facial color cue and luminance on people’s social perception [42,79]. Numerous researches have shown that evaluations of attractiveness could be influenced by the difference in skin color and condition [80]. Regarding the judgments of trustworthiness, researchers [42] have shown that cosmetics (vs. without cosmetics) could increase facial luminance and color cue, which, in return, improved the perception of likability, attractiveness, and trustworthiness. Similarly, researchers [79] suggest that color hue could have an impact on the evaluation of attractiveness in the face and healthiness in the skin.

To sum up, a social robot with followed internal features or its combinations might be considered as more trustworthy: round eyes, large eyes, direct gaze, brown eyes, short noses, upturned mouth, increased color cue, and luminance.

### 4.2. Implications for External Features

Face shape, including fWHR, brow-nose-chin ratio, and forehead-sellion-nose ratio, plays an important role in trustworthiness evaluation. Among those facial ratios, fWHR is the most prominent human secondary sexual characteristic and also the most commonly explored feature that could have an impact on social recognition in previous studies [25]. To be more specific, in human perception, large fWHR (vs. small fWHR) is perceived to be more dominant, aggressive, unattractive, and untrustworthy [29,44,56]. However, in the field of product evaluation, what might be counter-intuitive is a large fWHR of product design would be like more since it works as a signal of user’s dominant status (Detailed discussion in Section 4.5) [25]. Similarly, the brow-nose-chin ratio and forehead-sellion-nose ratio are negatively correlated with trustworthiness judgment [57]. However, the relationship between these ratios and trustworthiness might differ in various contexts. For example, the brow-nose-chin ratio is the only significant predictor for rating trustworthiness of 12-years old male’s face, however, it was not significantly correlated with other ages and another gender. Forehead-sellion-nose ratio was also the only significant factor for adult’s trustworthiness, but it was not significant for other scenarios.

There are several studies trying to explore other external features, influencing the judgment of trustworthiness [59,64,71]. Prior research has shown an ambiguous relationship between forehead size and trustworthiness. Based on the evidence from cross-cultural participants, prior research [71] suggested that taller and smaller (vs. shorter and bigger) forehead could contribute to trustworthiness evaluation. However, researchers [59] indicated that infants usually had a relatively prominent forehead, small chin, and short ears that implied trustworthiness based on evolutionary psychology [25,53]. The reason why this contradiction occurs might due to the different definitions of the same word. To be more specific, the word, “taller and smaller forehead”, previous research [71] mentioned refers to a relatively small area of a forehead (= width x height) with a relatedly long height. However, the forehead size mentioned in [29] is actually the distance from the eyes to the hair. Accordingly, the definition of forehead size and height needs to be explained more clearly in different contexts. Besides, Hellström and Tekle [49] suggested people with glasses (vs. no glasses) and a beard (vs no beard) were generally considered to be more helpful and trustworthy. In addition, hair (vs. bald) or absence of facial tattoos (vs. have facial tattoos) could contribute to the evaluation of good-looking, credibility, integrity, and leadership [46,81]. However, this effect relies on different occupations. For instance, salesman, who was typically considered with hair but no glasses, was strongly correlated with untrustworthiness, unintelligence, and suspect, in return, decreasing sales while highly educated people, such as professor, who were usually considered to wear glasses, a beard but no hair, were believed to be trustworthy, intelligent, and helpful [49,50]. 

Age and ethnicity also work as salient factors in facial trustworthiness [38,59,64,71,79]. There is a U-shape relationship between age and trustworthiness. Specifically, babyface (young age) and old face (old age) enjoyed the higher level of trustworthiness when compared with an adult face (middle age) due to the baby-face overgeneralization effect, a stereotype that children are unreliable witnesses [38,59]. Furthermore, although the evolution of signaling has shown human might consciously adapt visual cues or characteristics to emphasize or conceal heritable facial traits, influencing social perception and recognition, different ethical or cultural groups (e.g., Chinese vs. Canadian [42] or Caucasian vs. African vs. East Asian vs. South Asian [35]) tended to share and adopt similar facial cues to judge trustworthiness and attractiveness. However, some ethnic groups (e.g., Hungarians) [35,38] or their implicit ethical attitude [63] might be biased toward their own facial ethnicity.

In this way, a social robot with followed external features or its combinations might be considered as more trustworthy: large fWHR, small brow-nose-chin ratio and forehead-sellion-nose ratio, tall forehead, short ears, small chin, babyish looking, and consistent ethnicity.

### 4.3. Implications for Combinations of Features 

According to the baby-face overgeneralization effect (also called “baby schema”), people whose facial features have childlike traits (vs. without such traits) tended to have a high rate of cuteness and honesty, which are components of trustworthiness [57,58,59]. Typically, facial babyishness tended to have large eyes, a high brow ridge, a small chin, a pug nose, short ears, thin lips, and no-missing visible front teeth [25,56,57,59,69]. Despite all these social benefits, a “babyface” could be anticipated as being the opposite of dominance, namely, being considered as socially dependent, intellectually naive, and physically weak [25]. In accordance with a baby’s face, a feminine face (vs. masculine) usually shared similar facial traits, such as a bigger eye and short eye spacing [82,83]. Thus, people would believe masculine faces to be generally more dominant, less cooperative, and less honest while people would assume feminine faces to be more dependable, more cooperative, and more trustworthy [40,53]. 

In addition, people would have a high rating of trustworthiness towards those who looked similar to the perceivers, those who looked typical in perceivers’ cultural group or affiliation, those who previously presented before, and those whose face looked symmetrical [43,54,61,65,68,72]. The reasons lie in that both similarity and typicality could increase the familiarity that could eventually enhance positive evaluation of trustworthiness [43,61,65]. Exposure to socially relevant information could influence facial prototypes, shaping the unknown facial information processing, which mainly relies on the expectation of real-life experience. For example, our perceptions of strangers might be relied on the generalization of behavioral traits associated with previously seen facial features [54]. Furthermore, an evolutionary connection has well documented the relationship between symmetry in the face and trustworthiness since facial symmetry is a strong indicator of attractiveness and fitness [72]. When there exist hemifacial asymmetries, the left hemi-face (vs. right hemi-face) is responsible to communicate trustworthiness more efficiently in happy expressions because left hemi-face is associated with the emotional side of the brain (the right hemisphere), having the advantage to conceal anti-social intentions than right hemi-face [61].

### 4.4. Implications for Dynamic Features and Emotions 

Regarding the effect of facial movements on trustworthiness, previous studies have generally focused on the dynamic features of three regions: eye region movement, mouth region movement, and head movement. As discussed in Section 4.1, compared with eyes blink, eyes squint, and averted gaze, direct gaze might play a crucial role in attractiveness and trustworthiness evaluation since it could influence people’s attention and indicate social interest [33,52]. 

Similar to eye region, mouth movement also works as an effective predictor to communicate honesty and trustworthiness since it is strongly associated with positive or negative emotion expression, such as smiles [37,51,57,64]. Generally speaking, smiling is often associated with a U-shape mouth with raised lip corner and raised an eyebrow, indicating the related positive emotion expression, such as happiness. On the other hand, an inverted U-shaped mouth with the lower lip and lower eyebrow is often associated with sadness and anger [37,47,51,57]. Indeed, emotion and perceived trustworthiness interact with each other: while the happy face is considered more trustworthy, the trustworthy face is also believed to be happier [48,60,70]. Since the judgment of trustworthiness is often associated with happiness [33], mouth movement then seems to be a salient signal of social perception [37]. Although the smile is universally recognized as an indication of positive emotional experience, people could spontaneously notice different types of smiling, such as enjoyment/authentic smiles and non-enjoyment smiles, since different smiles might be associated with specific social meanings. Compared with non-enjoyment/fake smiles, people have a strong intention to trust and cooperate with people with enjoyment smiles [51,55]. 

Negative emotions, such as anger, disgust, or fear, might have a more nuanced effect on communicating credibility. They might be a context-related signal to communicate trustworthiness [48]. For example, the fear expression is characterized by raised inner and outer eyebrows, widened eyes, an outward pull of the lip corners, and dropped the jaw. When we evaluated them in the given context, for example, in the context of announcing alert message, Reed and DeScioli [84] have shown that people had a higher intention to believe this message with a fear expression, rather than a neutral expression, suggesting the negative expression could also add credibility in some cases, as positive emotion expression does. Another example could be found in evaluating criminal appearance ratings, Flowe [45] indicated angry expression would be perceived as less trustworthy and more dominant and even more criminal. However, when we evaluated negative emotions in a context-free scenario, fear expression might not significantly influence trustworthiness evaluation though angry or disgust expression still effectively contributed to untrustworthy perceptions [48].

Further, Engell and his colleagues [55] explored the scenario of how people evaluate the trustworthiness of neutral representations after initially adapting to a happy or angry face. Results showed initial adaptation to happy (or angry) expression would increase (or decrease) the perceived trustworthiness of neutral face in the later stage while fearful expression did not have such effect, suggesting a generalization effect that a common neutral system might be engaged when evaluating facial trustworthiness in angry or happy expressions. 

In addition to eye and mouth movement, other movements or responses could also lead to trustworthiness evaluation. For instance, facial blushing along with a head slightly downward movement usually indicates people concern about other opinions, feel sorry about their misdeed, and apologize in this non-verbal way [39]. A similar observation might also be seen in head nodding, which substantiates the reward power of facial cues in social interactions [70]. Accordingly, people with such embarrassment responses would like to be evaluated more positively and considered as more trustworthy. 

Consequently, a social robot might be perceived as more trustworthy if accompanied by the following dynamic and emotional features: to have a babyface, to have a symmetrical and feminine feature, direct gaze design, keep enjoyment smiles, or head nodding for the positive emotion.

### 4.5. Trustworthiness Evaluation in Human and Non-Human Perception

When making an evaluation of trustworthiness in humans and non-humans, people tended to rely on similar facial cues, such as eye shape, to make social perceptions [22]. However, when it comes to social robot design, selecting the appropriate set of facial characteristics from the previous human and non-human literature may not be simple. Indeed, there are several conflicts or inconsistencies in communicating trustworthiness worth noting and further examination. 

Previous research on the effect of fWHR on human trustworthiness has suggested humans with large fWHR (vs. small fWHR) are considered as more untrustworthy and unattractive [29,44]. However, Maeng and Aggarwal [25] have suggested that products with large fWHR (vs. small fWHR) are actually liked more, rather than less. The reason why people generally dislike dominant-looking human faces but like dominant-looking product appearance lies in that people could feel more arousal when faced with large fWHR products, thus enhancing and signaling their own dominant social status. On one hand, concerning the high association relationship between attractiveness and trustworthiness [57], it is reasonable to predict that large fWHR (vs. small fWHR) social robot might be generally regarded as more attractive and trustworthy. On the other hand, an appropriate match between a robot’s social cues and its task will improve people’s acceptance of and cooperation with the robot [85], it suggests that fWHR of social robots might depend on the roles of assigned tasks. For example, social robot such as an expert or a doctor that user would consider in a consultant role might be regarded as more professional if designed with large fWHR faces, whereas social robot such as a housekeeper that users wish to control over (e.g., like a servant) might be perceived as more trustworthy if endowed with small fWHR faces.

As stated in Section 4.4, typical looking people would be rated higher in trustworthiness because the typical face is extracted and averaged from faces previously seen (as more familiar) and serves as a standard against which all faces are evaluated in a given group or cultural affiliation [65]. However, when it comes to a social robot, it does not have a “typical” robot face since it is just an artificial machine without any heritable families. Although a social robot does not have a “typical” face, it would be interesting to explore whether a social robot face, adapted based on the principle of a typical human face of a given group, would also be treated as trustworthy accordingly. 

Another point worth mentioning is the Uncanny Valley effect [13,21,86]. It refers to the relationship between trustworthiness and likeness in a robot that does not follow a simple linear positive pattern: it might decrease when the artificial agent gets increasingly realistic but still have imperfect characteristics [86]. People would positively evaluate and interact with a robot when the robot looks like a human until a level beyond which people would suddenly show strong revulsion to the robot. As the appearance of a robot is increasingly made more human-like, people would gradually positively evaluate the robot again. Based on evaluating trustworthiness in 80 traditional humanoid robots, Mathur and Reichling [13] confirmed the existence of UV effect. However, different from traditional humanoid robots, social robots are designed with a screen to represent a ’face’ to dynamically communicate with users [87]. Regarding this, the user is actually interacting with an animated face, which is different from real facial features designed in humanoid robots [21]. The human facial resemblance degree (from animated face to artificial face to real face) in social robots might be the crucial point in addressing the nuanced effect on facial trustworthiness: the majority of people might find animated face trustworthy [12,21] while others tend to trust real human face (vs. artificial faces) [34]. Thus, it is theoretically and practically interesting to explore whether the UV effect still occurs within the domain of social robots.

## 5. Conclusions Remarks

Based on the systematic review on facial features from the human face, product appearance, and robot face, this paper evaluates and summarizes static facial features, dynamic features, their combinations, and related emotional expressions, shedding light on further exploration of facial trustworthiness for social robot design. 

**Figure 3:**
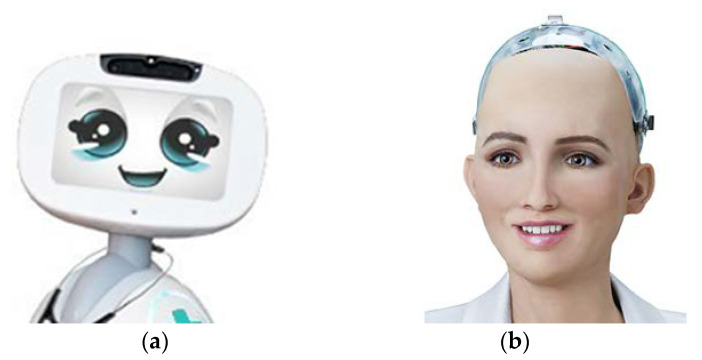
Trustworthy-looking robot models. (**a**) an animated face (Buddy Robot^®^) and (**b**) a realistic face (Sophia Robot^®^).

Concerning the results of the current systematic review, there are still some points that need to be acknowledged. To begin with, although we have discussed the potentially optimal features of facial trustworthiness in Section 4, we might still face an issue on how to integrate different features to create a harmonious face. Considering the abstract characteristics of an animated face in social robot [88], simply optimizing single features and combining all of them together does not necessarily make the whole face most trustworthy: creating a balanced trustworthy face is not an easy job and we might still take a risk of getting an uncanny “Frankenstein-like” face [86,89]. In order to address this problem, significant facial features, regions, and facial balance should be emphasized and given the priority for social robot design. Indeed, previous studies have suggested facial trustworthiness communication mainly depends on the interaction among static features, dynamic expressions, and general appearance characteristics [41]. As for static features, eye and face shape might be the most promising area because eye is a salient facial feature for catching people’s attention [52,57,59,64,67,71,77] while face shape (e.g., fWHR and forehead) is the most prominent human secondary sexual characteristics [25] and also the most obvious feature when evaluating a face [90]. Regarding dynamic facial features, the mouth region is the most pronounced feature for emotional expressions (happiness, anger, or sadness) due to its spontaneous muscle activity around mouth and lips [91]. With respect to the general appearance, babyface, which enjoyed the advantage of evolution and is characterized by the impression of extreme youth and innocence, might act as a significant factor communicating facial trustworthiness [31]. Another appearance concern that needs noticing is to avoid the negative influence of uncanny valley [13]. As Jentsch [86] indicated, “It is an old experience that the traditional, the usual, and the hereditary is dear and familiar to most people and that they incorporate the new and the unusual with mistrust, unease, and even hostility (misoneism)”. Under the existing level of technology, it might be not easy to create a highly realistic (three-dimensional or embodied) human-like robot [92]. Thus, it might be a smart and safe choice to create an animated (or photorealistic) face with certain human facial resemblance before the extent of resemblance could elicit unexpected negative reactions. Though it might be still difficult to determine the exact extent of facial resemblance, we have tried to take those factors into considerations to give a relative promising robot model. Figure 3 shows robot models (an animated face and a realistic face) with trustworthy-looking features. 

Since research on the facial design of the social robot is a multi-disciplinary field that is rarely systematically analyzed and sporadically studied by various fields, the current study has tried to systematically summarize potential features that could improve facial anthropomorphic trustworthiness for social robot. Future studies on human-robot trustworthiness might have the following research directions: theoretical exploration and empirical validation. As for theoretical exploration, a promising future study could try to explicitly discuss the theoretical foundation and evolution of trust in HRI since the theoretical works could also ground our comprehension of facial trust in HRI, which might not be the focus of the current study. In addition, the current study did not provide a robot model with dynamic trustworthy features. Concerning empirical validation, another promising stream of future studies could empirically examine the effect of different facial features on perceived trustworthiness from four main fields and compare their difference with human facial trust studies: (1) different shapes of eye and mouth in robotic face are essential internal features. Hence, they should be further validated the conclusions in human facial features; (2) WHR works as a salient trustworthy signal in human face. Thus, it would be theoretically interesting to compare the results between trust perceptions towards human and robot; (3) baby schema enjoys evolutionary advantages in human facial perception. Therefore, it would also be theoretically intriguing to verify whether it works in HRI; (4) future studies might also try to explore the effect of emotional expressions on trustworthiness and their interaction with different daily contexts, such as valence and arousal (also known as “urgency”) [93]. It would be both theoretically and practically significant to explore the interaction of emotional expressions and different daily contexts in influencing trustworthiness. Last, in order to have a more comprehensive illustration, a future study was planned to build a multi-media website to systematically illustrate trustworthy-looking robot models with static and dynamic features.

To sum up, since limited research has systematically provided specific guidance to help social robot designers and engineers improving trustworthiness in the robot’s face, future studies could try to obtain a holistic picture of trust in a social robot through a series of experiments, contributing to literature on HRI.

## Figures and Tables

**Table 1:** Summarized facial features on trustworthiness.

Database	Search Terms	Hits
Scopus (1960–2019)	Facial trustworthiness contained “(face OR facial) AND (trust OR trustworthiness* OR credibility OR trust traits* OR trust features* OR trust signs*)” Product or robot trustworthiness contained “(product OR robot OR anthropomorphism) AND (face OR facial) Trust”.	849
PsycInfo (1967–2019)		1214
Web of Science (1955–2019)		657

**Table 2:** Summarized facial features on trustworthiness.

Authors	Sample	Country	Application/ Purpose of study	Measure	Processing Technique	Results
Arminjon et al. (2015) [33]	57		To test the effect of lying cues (LC) in guessing behavior.	Yes or no proportion	Repeated measures ANOVA	Compared with NLC, LC was significant to lying decisions and is related to the automatic processing of lying detection.
Balas and Pacellaa (2017) [34]	51	US	To test the difference of trustworthiness perception between the artificial face and real face	1-7 Likert scale	T-test	Computer-generated faces were considered to be less trustworthy than real human faces
Birkás et al., (2014) [35]	266	US, Hungarian, East, and South Asia	To examine the effect of facial ethnicity on trustworthiness evaluation.	1-7 Likert scale	Two-way ANOVA	Different ethical groups showed similar trustworthiness evaluation. However, Hungarians tended to be biased toward their own ethnicity for medium/low trustworthy faces.
Brownlow (1992) [36]	128	US	To evaluate the difference in trustworthiness perception in baby-faced (vs. mature-faced) people	1-9 Likert scale	Three-way ANOVA	Baby-faced (vs. mature-faced) speakers enjoyed more positive trustworthiness evaluation and might induce more agreement when their trustworthiness was questioned.
Calvo et al. (2017) [37]	64	Spanish	To explore the effect of the combination of different mouth and eye on trustworthiness evaluation.	1-9 Likert scale; iNVT	Repeated measured ANOVA	Faces with an unfolding smile and eye looked more trustworthy. The contribution of the mouth was greater for happiness than for trustworthiness.
Cowell and Stanney (2005) [38]	45	US	To investigate the significance of face region in influencing the trustworthiness of anthropomorphic computer characters	1-7 Likert scale	A Kruskal–Wallis test	Face region plays a significant role in communicating trustworthiness, compared with the body region. Users tended to trust young-looking and ethnicity-consistent characters.
Dijk et al. (2011) [39]	196	Dutch	To explore the effect of blushing on trustworthiness.	Trust game choice; 1-7 Likert scale	Two-way ANOVA	The blushing people were perceived to be more trustworthy.
Dzhelyova et al., (2012) [40]	32		To test the relationship between trustworthiness and sex of face	Accuracy Rate	Mixed ANOVA	A female face would be perceived to be more trustworthy than a male face.
Engell et al., (2010) [41]	49	US	To investigate whether the previously perceptual similarities between trust and emotions (fear/happy) could extend to neutral representations.	1-9 Likert scale	Three-way ANOVA	Adapting to happy/angry faces could increase/decrease in the subsequent evaluation of trustworthiness in a neutral face.
Etcoff et al. (2011) [42]	149		To evaluate the effect of color cosmetics on trustworthiness.	1-7 Likert scale	A linear mixed-effects model	Cosmetics can exaggerate cues to sexual dimorphism, improving trustworthiness.
Farmer et al., (2013) [43]	59		To examine whether facial similarity could influence the judgments of trustworthiness and cooperative behavior.	Percentage of others’ face in the point of subjective equality	Repeated-measures ANOVA	Facial similarity has shown to have an effect on improving trustworthiness evaluation.
Ferstl et al. (2017) [44]	48		To explore the effect of facial features on the perceived personality and moral decisions.	1-7 Likert scale	A generalized linear mixed model	Human faces trustworthy traits might not be consistent with abstract faces.
Flowe (2012) [45]	512	UK	To investigate the relationship among perceived criminality, trustworthiness, facial mature, and emotional expression.	1-7 Likert scale	Two-way ANOVA	Angry faces are deemed as less trustworthy and more dominant.
Funk and Todorov (2013) [46]	286	US	To examine the effect of facial tattoos on perceived trustworthiness	1-7 Likert scale	Three-way ANOVA	Facial tattoos might lead to a lower level of trustworthiness evaluation.
Gill et al. (2014) [47]	12		To test the effect of phenotypic morphology on the default social traits.	1-5 Likert scale	Correlation Analysis	The facial movement could predictably modulate the perception of basic social traits in face morphology.
Gutiérrez-García and Calvo (2016) [48]	48	Spain	To investigate the relationship between trustworthiness and emotional facial expression	1-9 Likert scale	Three-way ANOVA	Trustworthiness is positively associated with the intensity of happy expression while negatively correlated with the intensity of angry and disgust face.
Hellström and Tekle (1994) [49]	75	Swedish	To evaluate the effects of different facial attributes (glasses, beard, and hair) on characteristic profiles.	1-6 Likert scale	Three-way ANOVA	The judges associated wearing glasses with intellectualism and goodness, being bald with idealism, and wearing a beard with unconventionality and goodness.
Jean François et al., (2013) [50]	180	Beguiler	To test whether hairstyle could influence trust detection.	Trust game; Money transfer rate	Three-way ANOVA	The hairstyle could influence people’s detection of trust.
Johnston et al. (2010) [51]	30	New Zealander	To investigate the effect of different types of smiling on attention.	1-7 Likert scale	Repeated-measures ANOVA	Enjoyment smiles are positively evaluated and are considered to have higher rates of cooperation.
Landwehr et al. (2011) [22]	263		To investigate the effect of product facial design on people’s liking.	1000 points scale	Repeated-measures ANOVA	Perception of friendliness is associated with the product with an upturned mouth, while aggressiveness is associated with the product with both an upturned mouth and slanted eyes.
Kaisler and Leder (2016) [52]	70	Austrian	To explore how eye contacting affects social and aesthetic evaluations.	1-7 Likert scale	Repeated-measures ANOVA	Direct-looking faces are considered to be more trustworthy.
Kleisner et al., (2013) [53]	238	Czech Republic	To test whether eye color influences the perception of trustworthiness.	1-10 Likert scale	A generalized linear mixed model	Brown-eyed faces were perceived as more trustworthy and the reason lies in the facial features associated with it.
Kocsor and Bereczkei (2016) [54]	116		To explore whether facial traits could have an impact on a composite face with such traits.	1-9 Likert scale	Chi-square test	Composite faces with high social desirability tended to be considered more trustworthy.
Krumhuber et al., (2007) [55]	90	UK	To examine whether facial dynamics could influence perceived trustworthiness and cooperative behavior	0-6 Likert scale	MANOVA	Authentic smiles enjoyed the highest level of perceived trustworthiness, followed by a fake smile and a neutral face.
Linke et al., (2016) [56]	187		To explore the relationship between facial geometric morphometrics and facial trustworthiness	1-7 Likert scale	Multivariate regressions	A trustworthy face might have lower fWHR, narrow lips, longer nose, larger eyes, and shorter eyebrows.
Luo et al. (2006) [21]	183		To investigate whether or not the on-screen characters representation influence trustworthiness perception.	1-7 Likert scale	One-way ANOVA and Paired t-tests	On-screen characters (OSCs) are considered to be more trustworthy in general. There is a mismatch between the expectations and capabilities of OSCs.
Ma et al. (2015) [57]	139	Chinese	To explore how children judge trustworthiness from faces	1-3 Likert scale	Stepwise linear regressions	8-years children could use a similar inference to evaluate trustworthiness. Different age groups could use different facial features to make an evaluation.
Maeng and Aggarwal (2018) [25]	248		To explore the face width-to-height ratio (fWHR) can signal dominance and affect its overall evaluation	1-7 Likert scale	A linear mixed-effects analysis using lme4 and lmerTest	High fWHR product is considered to be more dominant and liked more.
Maoz (2012) [58]	88	Israeli	To test the effect of babyface (vs. mature face) on politician trustworthiness evaluation	1-7 Likert scale	Two-way ANOVA	A baby-faced politician is believed to be more trustworthy (vs. mature face).
Masip et al. (2004) [59]	324	Spanish	To examine the impact of facial maturity on impressions of truthfulness.	1-7 Likert scale	MANCOVA	Baby-face and age are perceived to be a significantly static cue to make trustworthiness evaluation.
Mathur and Reichling (2016) [13]	334		To investigate whether human-robot interactions may be complicated by Uncanny Valley (UV)	Mean dollars wagered	Polynomial regression	The Uncanny Valley, in which imperfect human-likeness cues elicits dislike, could influence human perceptions of robots.
Oosterhof and Todorov (2009) [60]	60	US	To test the relationship between facial expression (anger and happiness) and perceptions of trustworthiness	1-8 Likert scale	Repeated-measures ANOVA	A trustworthy face with happy emotion was perceived happier than an untrustworthy face; an Untrustworthy face with angry emotion was perceived angrier than the trustworthy face.
Okubo et al. (2013) [61]	100	Japanese	To investigate the effect of a posed smile on people’s attitudes.	Response bias	Three-way ANOVA	The left–left composites were perceived to be more trustworthy when posed with a happy face.
Reed and DeScioli (2017) [62]	218		To test whether fear expressions add credibility to a speaker’s warnings of danger	1-7 Likert scale	Chi-square	Warning of danger with a fear expression is considered to be more trustworthy.
Stanley et al., (2011) [63]	50	US	To examine the effect of implicit ethical attitude on trustworthiness evaluation.	1-9 Likert scale	Stepwise regression analyses	Perceived trustworthiness towards people with various ethical racial backgrounds is associated with the extent of that individual’s implicit race bias.
Santos and Young (2011) [64]	Study 1: 24; Study 2: 48	UK	To investigate the importance of holistic processing in the inference of social attributes from faces.	1-7 Likert scale	Repeated-measures ANOVA	Experiment 1: internal features plays a more significant role in trustworthiness inferences. Experiment 2: different facial cues are used in different evaluations.
Sofer et al. (2015) [65]	53	Israel	To test whether face typicality is an important factor for social perception.	1-9 Likert scale	Repeated-measures ANOVA	For a continuum of faces that vary on a typicality-attractiveness dimension, trustworthiness evaluations peak around the typical face.
Stanton and Stevens (2017) [66]	52	Australia	To explore the relationship between gaze and trustworthiness evaluation	Mean answer change	Two-way ANOVA	People might trust the robot more on hard trials, compared with on medium trials. In addition, females are least likely to trust a robot that stared at them.
Stirrat and Perrett (2010) [29]	62	UK	To explore the effect of fWHR on trustworthiness evaluation	The proportion of trust in the image.	A least-squares regression	Wide face in men was perceived to be less trustworthy.
Todorov et al., (2008) [67]	21	UK	To examine the relationship between judgments of facial trustworthiness and approach/avoidance responses and approximate the valence evaluation	1-8 Likert scale	A least-squares regression	High inner eyebrows, pronounced cheekbones, wide chins, and shallow nose sellion looked more trustworthy
Verberne et al. (2015) [68]	111	Dutch	To examine the effect of facial similarity on trust evaluation.	1-7 Likert scale	A one-way MANOVA	As the rules in human similarity, the similarity in the virtual agent would also be considered as more trustworthy.
Willis and Esqueda (2008) [69]	200	US	To investigate the social consequences, such as trustworthiness evaluation, for individuals missing visible front teeth.	1-7 Likert scale	A one-way MANOVA	The absence of visible front teeth could decrease trustworthiness evaluation.
Wooddall et al., (1980) [70]	148	US	To test the role of visual cues in interpersonal trustworthiness	1-7 Likert scale	Mixed ANOVA	Smile and head nodes are strong indicators for trustworthiness evaluation.
Xu et al. (2012) [71]	144	Chinese and Caucasian	To explore the difference in the ethnical group in trustworthiness evaluation.	1-9 Likert scale	A least-squares regression	Chinese and Caucasian shared similar cues to make trustworthiness evaluation.
Zebrowitz et al. (1996) [72]	103	US	To investigate the effect of age on trustworthiness evaluation.	1-7 Likert scale	Correlation analysis	Babyfaceness, attractiveness, facial symmetry, and large eyes had a significant impact on trustworthiness evaluation.

Note: “Authors” refers to the author(s) of the specific article; “Sample” refers to the sample size used in the article; “Country” refers to the nationality of the sample in the article; “Application/ Purpose of study” refers to the research objective of the article; “Measure” refers to the measurement strategy conducted in the specific article; “Processing Technique” refers to the analytical method used in the article; “Results” refers to the relevant conclusion in the article.

**Table 3:** Summarized facial features on trustworthiness.

Static Features		Combinations	Dynamic Features	Emotions
Internal Features	External Features			
Eye size	fWHR	Baby-face (Cuteness)	Eye movement	Anger
Eye color	Brow-nose-chin (ratio)	Masculine/feminine	Mouth movement	Sadness
Eye shape	Forehead-sellion-nose (ratio)	Symmetry	Smile (Authentic/Fake)	Fear
Eye gaze	Hair	Look similar	Other movements	Happiness
Eyebrow	Forehead	Look typical		Disgust
Nose	Ears			
Mouth	Beard			
Lips	Chin			
Teeth	Glasses			
Cheek	Tattoo			
Color Cue	Age			
Luminance Contrast	Ethnicity			
